# Impacts of multiple anthropogenic stressors on the transcriptional response of *Gammarus fossarum* in a mesocosm field experiment

**DOI:** 10.1186/s12864-022-09050-1

**Published:** 2022-12-08

**Authors:** Marie V. Brasseur, Arne J. Beermann, Vasco Elbrecht, Daniel Grabner, Bianca Peinert-Voss, Romana Salis, Martina Weiss, Christoph Mayer, Florian Leese

**Affiliations:** 1 Leibniz Institute for the Analysis of Biodiversity Change, Zoological Research Museum A. Koenig, Adenauerallee 127, 53113 Bonn, Germany; 2 Aquatic Ecosystem Research, University of Duisburg-Essen, Universitaetsstrasse 5, 45141 Essen, Germany; 3 Centre for Water and Environmental Research (ZWU), University of Duisburg-Essen, Universitaetsstrasse 2, 45141 Essen, Germany; 4 Aquatic Ecology, University of Duisburg-Essen, Universitaetsstrasse 5, 45141 Essen, Germany; 5 Ennepe-Ruhr-Kreis, Hauptstraße 92, 58332 Schwelm, Germany; 6 Department of Biology and Environmental Science, Linnaeus University, Universitetsplatsen 1, 39231 Kalmar, Sweden

**Keywords:** Antagonistic stressor interaction, Flow alteration, Gene expression, Metabolic depression, Salinisation, Sedimentation, Transcriptomic stress

## Abstract

**Background:**

Freshwaters are exposed to multiple anthropogenic stressors, leading to habitat degradation and biodiversity decline. In particular, agricultural stressors are known to result in decreased abundances and community shifts towards more tolerant taxa. However, the combined effects of stressors are difficult to predict as they can interact in complex ways, leading to enhanced (synergistic) or decreased (antagonistic) response patterns. Furthermore, stress responses may remain undetected if only the abundance changes in ecological experiments are considered, as organisms may have physiological protective pathways to counteract stressor effects. Therefore, we here used transcriptome-wide sequencing data to quantify single and combined effects of elevated fine sediment deposition, increased salinity and reduced flow velocity on the gene expression of the amphipod *Gammarus fossarum* in a mesocosm field experiment.

**Results:**

Stressor exposure resulted in a strong transcriptional suppression of genes involved in metabolic and energy consuming cellular processes, indicating that *G*. *fossarum* responds to stressor exposure by directing energy to vitally essential processes. Treatments involving increased salinity induced by far the strongest transcriptional response, contrasting the observed abundance patterns where no effect was detected. Specifically, increased salinity induced the expression of detoxification enzymes and ion transporter genes, which control the membrane permeability of sodium, potassium or chloride. Stressor interactions at the physiological level were mainly antagonistic, such as the combined effect of increased fine sediment and reduced flow velocity. The compensation of the fine sediment induced effect by reduced flow velocity is in line with observations based on specimen abundance data.

**Conclusions:**

Our findings show that gene expression data provide new mechanistic insights in responses of freshwater organisms to multiple anthropogenic stressors. The assessment of stressor effects at the transcriptomic level and its integration with stressor effects at the level of specimen abundances significantly contribute to our understanding of multiple stressor effects in freshwater ecosystems.

**Supplementary Information:**

The online version contains supplementary material available at 10.1186/s12864-022-09050-1.

## Background

Freshwater ecosystems are globally affected by anthropogenic activities, leading to changes in physicochemical and hydromorphological conditions with deleterious effects on the associated biota [1]. Intensive land-use can be a major driver of running water degradation due to flow modifications, contamination with organic toxicants or high nutrient concentrations [2, 3]. Despite an increasing number of studies showing the negative impact of land-use on riverine systems [4–7], agricultural stressor effects are still difficult to predict, since they often coincide with a variety of other stressors such as climate stressors [8] or biological invasion [9]. Because rivers are highly connected ecosystems, the impact of anthropogenic activities is integrated across the whole catchment [10]. This leads to high temporal and spatial variations in stressor occurrences and complex interaction patterns, which are in addition mediated through food web interactions [11]. For example, decreased discharge due to water abstraction or damming directly affects the community composition of streams by decreasing species richness and shifting communities towards more tolerant taxa [12]. Because transport efficiency directly depends on flow velocity, effects of other stressors such as fine sediment deposition might be magnified indirectly [10]. Likewise, if more water evaporates during summer and is increasingly used for irrigation purposes, discharge decreases while salinity increases. Such stressor interactions lead to deviations of observed effects from the expected effects of the individual stressors. When combined stressor effects exceed the sum or product of single stressor effects (i.e. stronger effect on the response variable than predicted), they synergistically interact. Vice versa, when combined effects are lower than expected, these stressors show an antagonistic interaction (see [13] for a detailed discussion about the concepts).

Ecological and physiological models often explain deviations of multiple stressor responses from the expected additive effects through the activation of different protective pathways based on the limited energy budget available to an organism. If the evoked protective pathways are shared by two stressors, energetic costs can be mitigated, resulting in combined stressor effects which might be smaller than expected by an additive model (antagonism). If, however, distinct but dependent pathways are activated by the two stressors, energetic trade-offs can arise which result in higher stress responses than anticipated for an additive model [14].

In the present study, we focused on single and combined effects of reduced flow velocity, increased fine sediment load and increased salt (sodium chloride) concentration. These important agricultural and hydrological stressors often occur together and can therefore interact. Several studies aimed to disentangle single and combined effects of these stressors [6, 15, 16] or combined effects with other stressors [7, 17, 18]. These studies focused on community level effects, particularly on stream macroinvertebrates, which are important bioindicator organisms. For instance, reduced flow velocity was shown to decrease species densities and richness of EPT taxa [19]. Sediment erosion can result in macroinvertebrate community turnovers due to a loss of structural heterogeneity because open interstitial spaces are filled up [20, 21]. Increased ion concentrations due to salinisation occur in areas with high return flows from e.g. fertilisers, road salt, or from mining activities, but can also be the result of irrigation or of salt-water intrusions [22, 23]. Salinisation leads to altered chemical water conditions such as pH, affecting osmoregulation, oxygen consumption or growth rates in aquatic biota [24] and can result in community shifts towards more salt tolerant taxa [25].

The assessment of multiple stressor effects in communities is of central interest in ecological research. Often the change in abundance is the key parameter assessed. However, the time scales addressed in ecological studies are often days or weeks and organisms may withstand the stress conditions due to physiological acclimation for these short periods. Thus, despite strong physiological stress, the observed abundance data may not indicate the negative stressor effect. In contrast, changes in gene expression represent an immediate physiological response to environmental conditions and can reflect stressor effects on short temporal scales. Further, the transcriptional plasticity determines the limits of an organism’s ecological amplitude. Stressor effects exceeding this physiological compensatory mechanism are expected to be reflected on a higher ecological level (e.g. in altered species abundance or distribution range). Therefore, complementing observational data with gene expression data can provide a more holistic picture on stressor effects acting on biodiversity.

Here, we quantified the gene expression profile of a key freshwater macroinvertebrate species, the amphipod *Gammarus fossarum* Koch, in Panzer 1836 clade 11 [26] using RNA-sequencing (RNA-seq). *G*. *fossarum* prefers headwaters and upper reaches with higher flow velocities [27] and is sensitive to acidification, hypoxia and organic pollution [28]. Since these organisms often dominate stream invertebrate communities and possess an intermediate position in food webs [29, 30], a loss of these populations would have pronounced effects on stream ecosystems.

Based on the induced gene expression profiles in response to i) increased sodium chloride concentration (hereafter referred to as ‘salinity treatment’), ii) reduced flow velocity (‘flow treatment’) and iii) elevated fine sediment level (‘sediment treatment’), we assessed single and combined stressor effects using a mesocosm field experiment. Specifically, we aimed to:

1. Identify differentially expressed genes and molecular pathways involved in the response to single and multiple stressor exposure, and
2. Identify and quantify stressor interactions (synergistic/antagonistic).

We also compare the gene expression patterns to results obtained from abundance data generated in the same experiment [15] and discuss prospects and limitations of gene expression data for multiple stressor research on freshwater organisms.

## Results

Sequencing of the 64 transcriptome samples yielded 17,236,422 ± 2,005,378 (mean ± S.D.) paired-end reads, ranging from 13,283,344 to 22,704,046 read pairs. After homopolymer removal and quality filtering, 17,101,459 ± 1,989,353 reads per sample were retained. The *G*. *fossarum* transcriptome comprised 1,197,198 transcripts from 983,707 ‘genes’ with an E90N50 transcript length of 2,003 bp. All RNA-seq samples showed consistently high re-mapping rates (88.46 ± 1.84%). We detected 96% complete BUSCOs, indicating that the reference transcriptome was nearly complete (Table 1).

**Table 1:** Trinity de novo assembly metrics

Basic assembly statistics	
Trinity contigs (isoforms)	1,197,198
Trinity genes	983,707
Median contig length	354
Contig N50	855
E90N50 (25,952 genes)	2003
average remapping rate ± S.D. [%]	88.46 ± 1.84
BUSCO	C:96.4% [S:38.1%, D:58.3%], F:0.7%, M:2.9%

BUSCO analysis was performed with the arthropod BUSCO dataset (1,013 BUSCOs); *C* = Complete, *S* = Single, *D* = Duplicated, *F* = Fragmented, *M* = Missing

### Increased salinity induces the strongest transcriptomic response

**Fig. 1:**
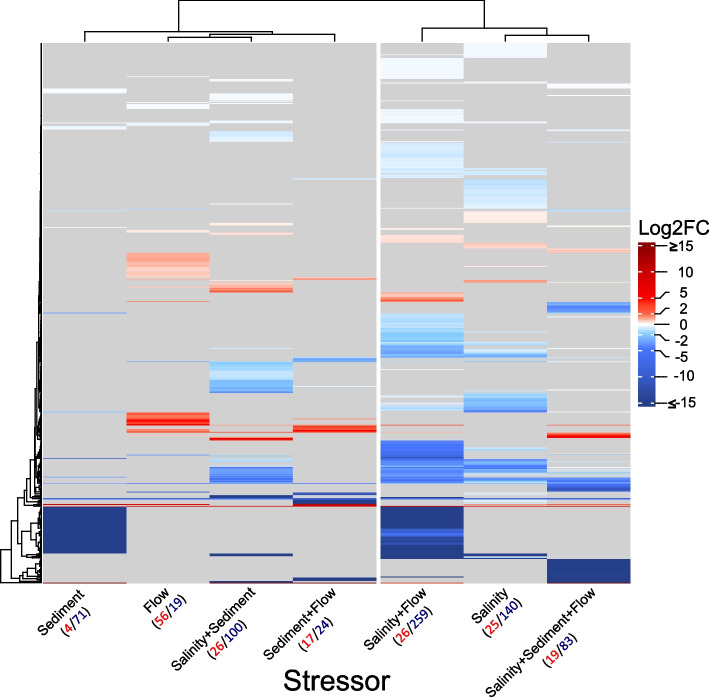
Heatmap showing the differential expression of genes in response to single and combined stressor exposure. Grey bars indicate changes in expression, which are not significant (adjusted *p*-values ≥ 0.1). Numbers in red and blue denote upregulated and downregulated genes, respectively

After gene level summarisation and filtering, 73,479 genes were retained and tested for differential expression. Across all treatment combinations, 613 genes were identified as differentially expressed. Overall, the expression profiles were dominated by downregulation of the affected genes (Fig. 1).

**Fig. 2:**
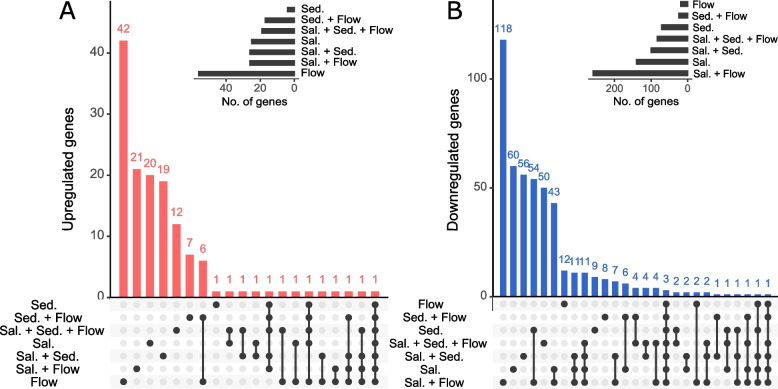
UpSet plots, summarising unique and shared differentially expressed genes due to exposure to different treatments. Black dots in panel matrices correspond to different Venn diagram sections: connected dots indicate intersections of sets of differentially expressed genes; singular dots represent unique sets of differentially expressed genes. Vertical barplots summarise the number of upregulated (a) and downregulated (b) genes. Horizontal bars indicate the total number of regulated genes in each treatment combination. The set union of all upregulated and downregulated genes detected in salinity treatments represents 443 salinity genes, of which one gene was upregulated when increased salinity was combined with reduced flow but downregulated when all three stressors were applied (= 442 exclusively regulated genes)

Only under reduced flow velocity, more upregulated (56 genes) than downregulated (19 genes) genes were detected (Fig. 1) and the highest number of exclusively upregulated genes was detected for this treatment (Fig. 2). Reduced flow velocity in combination with increased sediment levels resulted in the lowest number of differentially expressed genes (41 genes), followed by the single stressor treatments low flow velocity and increased fine sediment (both induced the differential regulation of 75 genes).

However, in combination with increased salinity, reduced flow velocity induced the strongest gene regulatory response (285 genes of which 139 were exclusively detected in this treatment combination). Interestingly, 54 of the downregulated genes in the salinity + flow treatment were also differentially downregulated when only fine sediment was added but not in any of the other stressor combinations (Fig. 2). Treatments involving increased salinity had the most pervasive effects on the gene expression profile: a total of 528 genes (86%) responded in the treatments that included increased salinity, of which 442 were exclusively regulated under increased salt concentrations. All salinity treatments, except the combination of increased salinity and added fine sediment, formed a cluster based on Euclidean distances obtained from shrunken LFC values (Fig. 1).

### Stressor interactions are mainly antagonistic

For 139 differentially expressed genes, a significant interaction was detected in at least one treatment combination (see Additional file 1, Table S1). Due to the interaction of increased salinity with reduced flow velocity, 109 genes responded differently than expected based on the individual effects (Table 2). When increased salinity co-occurred with added fine sediment, 31 genes showed a significant interaction. Under combined stressor exposure of reduced flow velocity and elevated fine sediment, 27 antagonistic gene expression patterns were identified. Finally, we found 7 genes that showed a three-way interaction when all stressors were applied (Table 2).

**Table 2:** Number of genes, which have an additional LFC due to the interaction between stressors

Stressor interaction	Salinity*Flow	Salinity*Sediment	Sediment*Flow	Salinity*Sediment*Flow
Positive synergistic	-	-	-	-
Negative synergistic	13	-	-	-
Positive antagonistic	85	3	7	2
Negative antagonistic	11	28	20	5
Sum	109	31	27	7

**Fig. 3:**
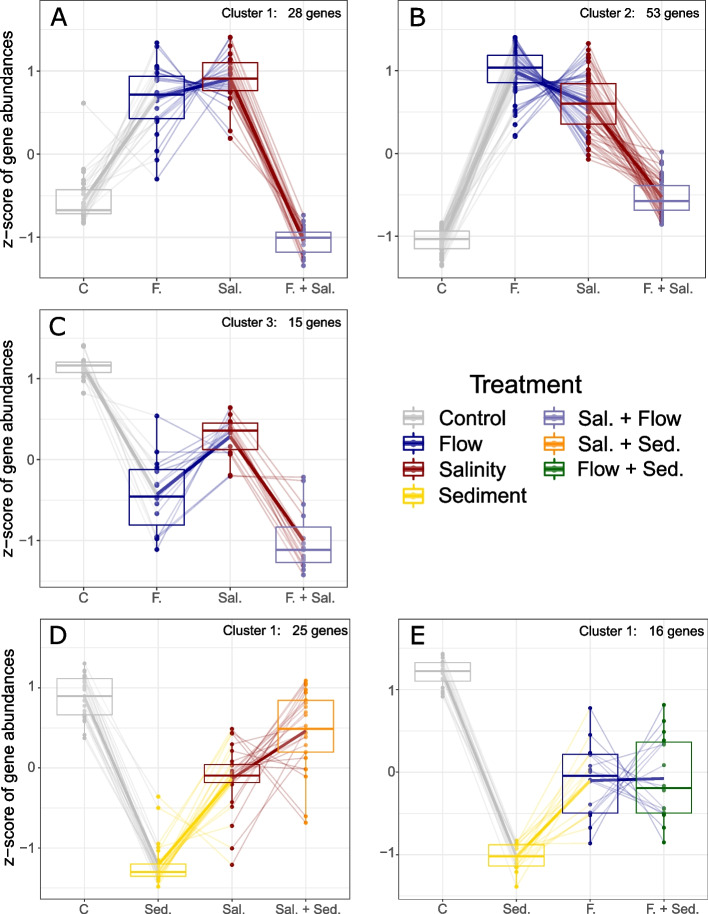
Clustering of genes, which showed an interaction between two stressors. The expression significantly differed from expectations based on the single stressor effects due to the interaction between salinity and flow (a-c), sediment and salinity (d) or sediment and flow (e). The z-score represents the scaled and centred gene expression. Positive and negative z-scores indicate expression values above and below the average across samples, respectively. Pale lines connect expression values of individual genes, bold lines the average expression values

Clustering of genes that showed an interaction between increased salt concentration and reduced flow resulted in three distinct groupings (Fig. 3a-c). The first two clusters were characterised only by A + interactions, i.e., genes that were less upregulated than expected due to the combined effect of reduced flow and increased salinity. The third cluster comprised genes, which were either stronger (S-) or weaker downregulated (A-) than predicted based on single stressor effects. Likewise, the two clusters formed by genes which responded differently to added fine sediment when either increased salinity (Fig. 3d) or reduced flow velocity (Fig. 3e) were applied, encompassed only A- interactions. In these cases, the downregulation of genes induced by added fine sediment decreased when this stressor was combined with one of the other stressors.

### Metabolic suppression appears to be a physiological stress response in *G*. *fossarum*

Transdecoder predicted 269,426 coding sequences, which were included in the annotation. In total 43,826 genes were successfully annotated, corresponding to only 5% of the whole assembly. From the set of genes that were included in the differential expression analysis, 20% were annotated with GO terms.

All stressor treatment combinations except the reduced flow velocity treatment induced a strong metabolic suppression in *G*. *fossarum.* The downregulated genes were annotated with GO terms suggesting gene involvement in biosynthetic processes, organisation of cellular components or inter- and intracellular signalling. While traversing the DAG towards more specific GO terms, it became evident that these terms either refer to (i) energy metabolism (e.g., GO:0045333, ‘cellular respiration’; GO:0046034, ‘ATP metabolic process’) or (ii) regulation of gene expression and polypeptide synthesis (e.g., GO:0043043, ‘peptide biosynthetic process’; GO:0043604, ‘amide biosynthetic process’). On the contrary, terms referring to the organonitrogen metabolism were annotated to upregulated genes in the reduced flow velocity treatment.

This stressor induced metabolic suppression was further supported by the functional enrichment analyses. Genes downregulated due to fine sediment addition, increased salinity, fine sediment addition in combination with reduced flow or increased salinity in combination with reduced flow were enriched for mitochondrial processes and ATP synthesis, as well as catabolic processes (e.g., GO:0005980, ‘glycogen catabolic process’; GO:0006635, ‘fatty acid beta-oxidation’). Linked to the lowered metabolic activity, we detected several functionally enriched GO terms referring to reproduction or growth in response to reduced flow velocity (GO:0022414, ‘reproductive process’) and reduced flow in combination with added fine sediment (GO:0008340, ‘determination of adult lifespan’). Many overrepresented GO terms comprised biological processes involved in DNA organisation, transcription and translation. These terms were enriched in the sets of genes downregulated in all salinity treatments and in response to increased fine sediment alone (see Additional file 1, Table S2).

Additionally, the gene expression profiles were characterised by downregulation of cellular stress responses (e.g. GO:0006457, ‘protein folding’; GO:0034605, ‘cellular response to heat’), which were attributed to the downregulation of putative heat-shock proteins (HSPs) in response to increased fine sediment or increased salinity in combination with reduced flow velocity.

The functional enrichment analyses testing salinity-specific genes further supported the general stress response involving metabolic processes, protein biosynthesis and cellular stress responses (see Additional file 2, Fig. S1, S2).

The genes which showed a negative synergistic interaction comprised a component of the mitochondrial cytochrome b-c1 complex (TRINITY_DN186_c1_g1) and the HSP83 (TRINITY_DN179_c0_g1). Further, three genes were annotated as uncharacterised proteins of which one likely represents a further chaperone (TRINITY_DN314351_c0_g1) and three appear to be involved in protein biosynthesis (TRINITY_DN14015_c3_g1, TRINITY_DN15558_c0_g1, TRINITY_DN315110_c0_g1).

## Discussion

Most stressor treatments resulted in a strong downregulation of the majority of genes in *G*. *fossarum*. When confronted with unfavourable environmental conditions, organisms are expected to allocate energy from processes like reproduction, growth or locomotion to vitally essential processes. This is supported by the downregulation of genes involved in these metabolic processes in response to the stressor treatments in the present study. It might seem counterintuitive that many downregulated genes were functionally enriched for biological processes involved in protein biosynthesis. In fact, processes involved in gene expression such as mRNA splicing are energetically costly since the polymerisation of pre-mRNA and the subsequent processing of RNA by the spliceosome is ATP-dependent [31]. When organisms are confronted with adverse habitat conditions, the energetic trade-off between transcriptional plasticity and suboptimal overexpression must be minimised in favour of maintaining homeostasis. Our findings imply that this could comprise cellular stress compensatory mechanisms such as the expression of HSPs. HSPs are not only relevant for protein refolding under cellular stress but are essential for folding newly synthesised proteins. When the stressor induced metabolic suppression leads to a general decrease in protein biosynthesis, less HSPs are required to counteract cellular proteotoxic stress.

### Single and combined stressor effects

Added fine sediment and reduced flow velocity induced the lowest number of differentially expressed genes. *Gammarus* tends to avoid habitats with increased fine sediment load, reflected in decreased abundances and increased drift propensities of these organisms at elevated sediment levels [15] and were shown to prefer habitats with coarse substratum over less complex habitats characterised by sand [32, 33]. If gammarids can inhabit stream bed habitats with their preferred particle size, they require less oxygen [34] and show increased growth rates [33]. During periods with high energetic maintenance costs, less energy can be allocated to processes, which are not required to maintain homeostasis. Our results suggest that this is at least partly controlled via sediment-induced energetic downregulation of specific metabolic genes and genes associated with protein biosynthesis. A similar metabolic depression was detected in other invertebrates like mussels [35] or shrimps [36] exposed to abiotic stress such as temperature, indicating that a reduction of cellular metabolic activity is a general strategy of different organismic groups to cope with stressful conditions.

In contrast, reduced flow velocity induced gene expression as this was the treatment with the highest number of upregulated genes. Compared to other native *Gammarus* species such as *G*. *pulex* or *G*. *roeselii*, *G*. *fossarum* is known to inhabit faster flowing stream sections [27] but habitat preferences are often linked to several factors such as reduced interspecific competition or risk of predation [12]. In fact, gammarids were shown to prefer moderate velocities, probably due to increased energetic costs associated with high flow velocities [32]. Gammarids have been found to be more active under lower flow velocities [37], which might reflect that more energy can be allocated to locomotion under moderate flow velocity.

Increased salinity alone and in combination with other stressors induced the largest number of differentially regulated genes in *G*. *fossarum*. Salt stress is known to regulate the expression of enzymes involved in detoxification of superoxide radicals e.g. superoxide dismutases or glutathione S-transferases [38, 39]. We found two putative glutathione transferase enzyme genes, which were exclusively regulated under increased salinity concentrations (namely TRINITY_DN1113_c0_g1, upregulated when only salinity was increased and TRINITY_DN613487_c0_g1, downregulated when increased salinity was combined with reduced flow velocity). Further, salinity stress induces oxidative stress in mitochondria of aquatic organisms through the production of reactive oxygen species [38]. In the present study, several GO terms were enriched for molecular functions involved in ATP metabolisms and one biological process involved in mitochondrial double-strand repair mechanisms in the set of salinity-specific genes. Freshwater crustaceans are hyperosmoregulators that maintain extracellular and intracellular ion homeostasis against an osmotic gradient. The passive loss of salts is regulated through expression of ion transporters like the Na^+^/K^+^-ATPase [40] or Na^+^/K^+^ /2Cl^−^ co-transporters [41]. Changes in the expression of genes encoding these ion transporters were for example reported for *Daphnia* during salinity stress [31]. In our study, two genes which are annotated to encode subunits of the Na^+^/K^+^-ATPase were downregulated in response to salinity and flow or when all three stressors were applied (TRINITY_DN67768_c1_g1, TRINITY_DN103746_c0_g1), but also in treatments involving sediment and flow manipulations (TRINITY_DN103746_c0_g1). The differentiation between metabolic processes involved in particular physiological stress response or those involved in general energy allocation requires further research. This is especially important for a deeper understanding of multiple stressor interactions when using RNA-seq data.

### Comparison to stressor effects derived from abundance data

In our experiment, the fine sediment induced expression profile disappeared when flow velocity was additionally reduced. This compensatory mechanism is consistent with the observations made by [15] based on abundance data, which showed that gammarids were more abundant under low flow conditions and their drift propensities decreased. The transcriptional activity of *G. fossarum* indicates that reduced flow velocity is tolerated by gammarids, or even preferred under the experimental conditions. Under natural conditions, other physicochemical parameters such as oxygen content or water temperature change during low flow periods. For example, gammarids seek for drying refugia within the interstitial spaces in the streambed when streams become intermittent. If fine sediment load prevents the hiding in the interstitial or hamper vertical movement of organisms, as found by [42], the combined effects of increased fine sediment and reduced flow velocity are expected to be stronger in natural streams. Further, the transcriptional profiling identified increased salinity as the most pervasive stressor. This is opposed to the patterns obtained from abundance data from the same experiment [15], where no effect due to salinity was detected for gammarids, neither in single nor in combined stressor treatments. The high temporal resolution of expression data provides insights in organismic stress responses which are not indicated by abundance data and an integration of these ecological endpoints provide a more holistic picture about multiple stressor effects. If salinity remains increased but gammarids are unable to maintain the physiological stress response for a longer period of time, drift and mortality rates are expected to increase. If the metabolic suppression can be maintained by *G*. *fossarum* in order to cope with increased salinity and the related cellular toxic effects, then this probably affects specimen body sizes, biomass, and fecundity. Therefore, irrespective of whether or not the stress induced physiological state can be maintained, the long-term effect of increased salinity is likely to have adverse consequences on the whole population.

We detected many more non-additive interactions than observed in multiple stressor effects based on specimen abundances in [15]. Inherently, this is related to the larger number of tested response variables (i.e., genes) compared to community data, reflecting the fine resolution of transcriptomic data on both temporal and mechanistic scales. The fact that these interactions are mostly antagonistic shows that the observed expression patterns do not predictively scale along intensifying stressor gradients. The self-regulatory plasticity of the transcriptome, which involves many regulatory pathways, signal transduction cascades and posttranscriptional or posttranslational modifications appear to play a fundamental role in physiological coping strategies.

### Limitations of transcriptomic profiling in non-model organisms

RNA-seq has become a powerful tool to study gene regulatory mechanisms. Although it is nowadays commonly used in biological sciences, most applications are still limited to biomedical sciences or when using highly controlled experimental setups. Under experimental conditions with many degrees of freedom (as in our mesocosm experiment, which aims to reflect near-natural conditions), an accurate detection of a true biological signal is more challenging because gene dispersion estimates will be inflated due to introduced variation that is unrelated to the tested treatments, preventing these genes to be detected as differentially regulated. The low signal-to-noise ratio, which is a common problem for RNA-seq data [43, 44], can not only have statistical but also biological or technical reasons. For example, very distinct transcriptomes will be sequenced together when working with non-model organisms collected from the field that are genetically highly diverse. If a reference genome is absent, the bioinformatic processing (especially the de novo assembly and subsequent mapping of reads) of these sequencing libraries can yield inadequate gene abundance estimations due to over-splitting of genes into too many contigs. Functional transcriptomic profiling in non-model species such as *G*. *fossarum* is further complicated by the scarce annotation data. Crustaceans are highly underrepresented in genomic resource databases, as has also been found by [45, 46]. In the current study, only 20% of the genes that were tested for differential expression were annotated. Assuming that more conserved genes will be more often annotated because their annotation appears to be more reliable, functional enrichment tests tend to identify more conserved gene functions, although this is not necessarily the main underlying physiological response. This highlights the need for ongoing research and effort in the field of functional genomics in non-model organisms.

## Conclusion

Anthropogenic stressors, like salinisation, an elevated fine sediment level or reduced flow velocity, degrade stream ecosystems and negatively affect key macroinvertebrates such as *G*. *fossarum*. We found a reduced transcriptional activity of many genes in response to stressor exposure, probably due to increased energetic maintenance costs. The compensatory effects between added fine sediment and reduced flow velocity are consistent with specimen abundance and drift data, indicating that RNA-seq data can detect stressor effects, which are similarly propagated to higher ecological levels. The transcriptomic profiling further indicated that increased salinity was by far the most pervasive stressor at the physiological level. This, however, is not reflected in abundance data of gammarids, suggesting that the differential downregulation due to increased salt concentration is part of a physiological acclimation strategy, which enables *G*. *fossarum* to remain at affected sites. Our data show that transcriptomic data provide important information and enhance our understanding of multiple-stressor effects from genes to ecosystems.

## Methods

### Experimental setup

To identify and disentangle the individual and combined effects of the stressors increased salinity, reduced flow velocity and elevated fine sediment levels, an ExStream mesocosm field experiment [18] was conducted at the Felderbach (Germany, North Rhine-Westphalia, 51°20′59.09″N, 7°10′14.03″E, 136 m a.s.l) from 8 March to 22 April 2014 (a full experimental description is given in [15]). Stream water was pumped in four header tanks for 46 days (24-day colonisation period, 22-day manipulative period). Each header tank supplied water to 16 mesocosms (diameter 25 cm, volume 3.5 l) via gravity. This ensures the same light regime, water temperature and chemistry in the experiment and the natural stream system. All mesocosms were filled with 300 ml fine sediment (< 2 mm), 900 g gravel (2–30 mm), 4 stones (> 30 mm) and three big stones, reflecting the natural composition of the streambed, and two leaf litter bags filled with 2.5 g dried alder leafs. Colonisation of mesocosms by stream organisms with a diameter < 4 mm occurred passively via drift and was complemented by macroinvertebrates that were actively collected in the stream one week prior to the start of the manipulative period. During the manipulative period, stream organisms were exposed to multiple stressor combinations in a 2x2x2 factorial design with eight replicates per treatment, comprising three factors (salinity, fine sediment, and flow velocity) with two levels each: ambient salinity (18.2 mg/l, SD ± 4.1) versus increased salinity (312.2 mg/l, SD ± 78.5) in terms of chloride concentration, natural flow velocity (16.5 cm/s, SD ± 0.1) versus reduced flow velocity (9.6 cm/s, SD ± 0.1) and ambient levels of fine sediment (300 ml, < 2 mm) versus added fine sediment (750 ml, < 2 mm). The chosen stressor conditions for flow velocity and sediment cover were shown to be realistic for streams in agricultural areas and the increased salinity concentration is expected to be reached based on chloride thresholds implemented into german law (see [15] for further details).

At the end of the experiment, 145 amphipods (2–4 specimens per mesocosm) were sampled from the channel substratum, irrespective of their sex or life stage. The organisms were snap frozen in liquid nitrogen and stored at -80 °C until nucleic acid extraction.

### RNA extraction, library preparation and sequencing

Frozen samples were disrupted with a disposable pestle and homogenised in 500 µl TRIzol reagent, allowing the dissociation of nucleoprotein complexes. After incubation, 200 µl chloroform were added and samples were centrifuged. RNA was isolated from the aqueous phase and precipitated with absolute ethanol. RNA was then purified using the RNeasy Plus Micro Kit (QIAGEN). Briefly, the samples were transferred to RNeasy mini-spin columns and washed with 700 µl RW1 buffer, followed by a wash step with 500 µl RPE buffer and finally with 500 µl 80% ethanol. The RNA was eluted in RNase-free water and sample integrity and concentration were assessed with a Fragment Analyzer using the RNA 15 nt kit (Agilent), following the manufacturer instructions. Based on sample quality, the extracts of two individuals per experimental unit (channel) were pooled for sequencing. For two channels, only one specimen was used. Library preparation and sequencing was done separately for each experimental unit, resulting in 64 RNA-seq libraries. RNA extracts were sent to Macrogen and cDNA libraries were prepared with the TruSeq RNA Sample Prep v2 kit. Libraries were 150 bp paired-end sequenced on an Illumina NovaSeq 6000.

### DNA extraction and barcoding

DNA was isolated from the lower organic phase and ethanol precipitated. The DNA pellet was washed 1–2 times with 500 µl 0.1 M sodium citrate in 10% ethanol (pH 8.5), followed by a final wash step with 1 ml 75% ethanol. The DNA was resolubilised in 150–300 µl 8 mM NaOH and pH was adjusted to ~ 8 using HEPES.

Species identity of amphipods was confirmed via DNA barcoding. The 658 bp barcoding fragment of the cytochrome-c-oxidase subunit 1 (COI) gene was amplified using the primer pair LCO1490-JJ/HCO2198-JJ [47]. Per reaction, 2.5 μl buffer (10x), 2.5 μl dNTPs (2 mM), 2.5 μl MgCl_2_ (25 mM), 0.125 μl HCO2198-JJ (100 μM), 0.125 μl LCO1490-JJ (100 μM), 0.2 μl *Taq* polymerase (5 U/μl, VWR), 14.04 μl PCR water and 1 μl DNA were used. PCR cycling conditions were 2 min of initial denaturation at 94 °C, followed by 33 cycles of denaturation at 94 °C for 20 s, annealing at 50 °C for 30 s, elongation at 72 °C for 1 min, and a final elongation at 72 °C for 5 min. PCR products were purified for Sanger sequencing using the exonuclease *ExoI* (20 U/μl, Thermo Scientific™) and alkaline phosphatase *FastAP* (1 U/μl, Thermo Scientific™). Bidirectional Sanger sequencing was performed at Eurofins Genomics. COI barcodes were compared to NCBI (www.ncbi.nlm.nih.gov/) and only individuals identified as *G*. *fossarum* (n = 130) were used for sequencing, irrespective of the mitochondrial clade they were assigned to.

### Assembly & read abundance estimation

Raw reads were homopolymer trimmed with a custom C +  + script and quality trimmed with the cutadapt v 3.2 [48] wrapper script TrimGalore! v 0.6.6 (https://github.com/FelixKrueger/TrimGalore) in paired-end mode with a quality cut-off Phred > 20, retaining only reads with a minimum length of 25 bp. De novo assembly of reads was performed with Trinity v 2.9.0 [49], based on four library replicates per treatment. To reduce redundancy in the transcriptome, contigs were clustered at 96% similarity with CD-HIT-EST v 4.8.1 [50]. Transcript abundances were estimated via mapping of quality trimmed reads against the clustered transcriptome using RSEM v1.3.3 [51] with bowtie2 v 2.3.5.1 [52] as mapper. Assembly quality and completeness was assessed by searching for arthropod benchmarking universal single-copy orthologs (BUSCOs) (BUSCO v 4 arthropoda_odb10, comprising 1,013 BUSCOs, [53]). Furthermore, we determined remapping rates of all sequencing libraries (8 libraries per treatment) and the corresponding E90N50 statistics, which represents the N50 value for the subset of genes that account for 90% of the total expression.

### Statistical analyses

Estimated transcript counts were summarised to gene level with the R package tximport [54], adjusting isoform abundances according to gene length. For gene inferences, we used the Trinity assumptions about isoform-gene relations. Since no reference genome for *G*. *fossarum* is available, the term ‘gene’ is used loosely here. Only genes with at least 10 normalised counts in at least 8 samples were included in downstream analyses. Gene counts were modelled with the R package DESeq2 [55] using the design ~ Salinity*Sediment*Flow. To account for hidden unwanted variation, surrogate variables were constructed from normalised counts (full model: ~ Salinity*Sediment*Flow, reduced model: ~ 1) using the sva package [56]. All significant surrogates were incorporated in an updated model as covariates.

Statistical inferences of individual and combined stressor effects were conducted using the Wald test statistic, applying a significant threshold of < 0.1 after FDR correction for multiple testing. LFC values were shrunken with the adaptive shrinkage estimator from the ashr R package [57].

To identify stressor interactions, we classified interactions as synergistic if the combined stressor effects were larger, and as antagonistic if they were smaller than the product of the single stressor effects. More specifically, a positive synergistic interaction (S +) is found when the observed gene expression was more positive (stronger upregulated) than expected; a negative synergistic (S-) interaction indicates that a stronger downregulation is observed than expected compared to the addition of main effects. Positive antagonistic (A +) interactions are defined as ‘less upregulated than expected’, whereas a negative antagonistic (A-) interaction refers to genes that are ‘less down regulated than expected’ due to the interaction effect.

### Functional annotation and enrichment

TransDecoder (https://github.com/TransDecoder/) was used to identify coding DNA sequences and the putative peptides were searched for protein domains using hmmer v3.3 (http://hmmer.org/) and the Pfam database [58]. Further, they were queried against the Swissprot/Uniprot database [59] using blastp [60] with 1e-5 as e-value cut-off. Homology inferences were integrated in the final prediction of proteins.

Predicted proteins were iteratively annotated via blastp (e-value 1e-5) searches against (i) the *Daphnia pulex* proteome (similarity ≥ 40%) (http://wfleabase.org) and (ii) the invertebrate Uniprot Swissprot/TrEMBL database (similarity ≥ 50%). Annotations from the invertebrate database were only included if no annotation was obtained from the *D*. *pulex* proteome, and the best protein hit (highest bitscore) was retained.

Gene ontology (GO) terms [61, 62] were obtained from UniProt IDs of proteins that were successfully mapped to isoforms. To obtain a general overview about the annotation data set, ancestor terms were derived from the GO.db [63] R package and sorted according to their position in the directed acyclic graph (DAG) with GOxploreR [64]. Functional enrichment analyses of GO terms associated with biological processes were performed with topGO [65] using Fisher’s Exact test and the weight01 algorithm. All genes which were tested for differential expression were used as gene universe and enrichment analyses were performed separately for up- and downregulated genes. In order to test for enriched GO terms in the set of genes which were exclusively regulated due to increased salinity (hereafter referred to as ‘salinity specific’), GO enrichment analyses for biological processes and molecular functions were performed using all genes which were exclusively differentially expressed in response to increased salinity, either in a single or combined stressor treatment.

### Clustering and visualisation

Genes which showed significant interactions between two stressor treatments were clustered according to their expression profiles using DEGreport [66]. The variance stabilised counts of these genes were corrected with a frozen surrogate variable analysis [56] and the minimum cluster size was set to 10. Significant clusters of expression profiles and GO term enrichment results were visualised with ggplot2 [67]. Upset barplots were generated with UpSetR [68]. Heatmaps were created with functions from the R package ComplexHeatmap [69] and dendrograms were based on Euclidean distances obtained from shrunken LFC values.

## Supplementary Information

**Additional file 1.**

**Additional** **file** **2.**

## Data Availability

The RNA-seq libraries have been deposited in the European Nucleotide Archive (ENA) at EMBL-EBI under project accession number PRJEB56296.
