# Supplementary material for: Impacts of multiple anthropogenic stressors on the transcriptional response of Gammarus fossarum in a mesocosm field experiment
Source: BMC Genomics. 2022 Dec 8;23:816. doi: 10.1186/s12864-022-09050-1 (PMC9733165; doi:10.1186/s12864-022-09050-1)
Supplement: Supplementary file 2 — Additional file 2. [file 12864_2022_9050_MOESM2_ESM.docx]

Additional file 1 for

**Impacts of multiple anthropogenic stressors on the transcriptional response of *Gammarus fossarum* in a mesocosm field experiment**

Marie V. Brasseur*, Arne J. Beermann, Vasco Elbrecht, Daniel Grabner, Bianca Peinert-Voss, Romana Salis, Martina Weiss, Christoph Mayer, Florian Leese

*Corresponding author: Marie V. Brasseur (m.brasseur@leibniz-lib.de)


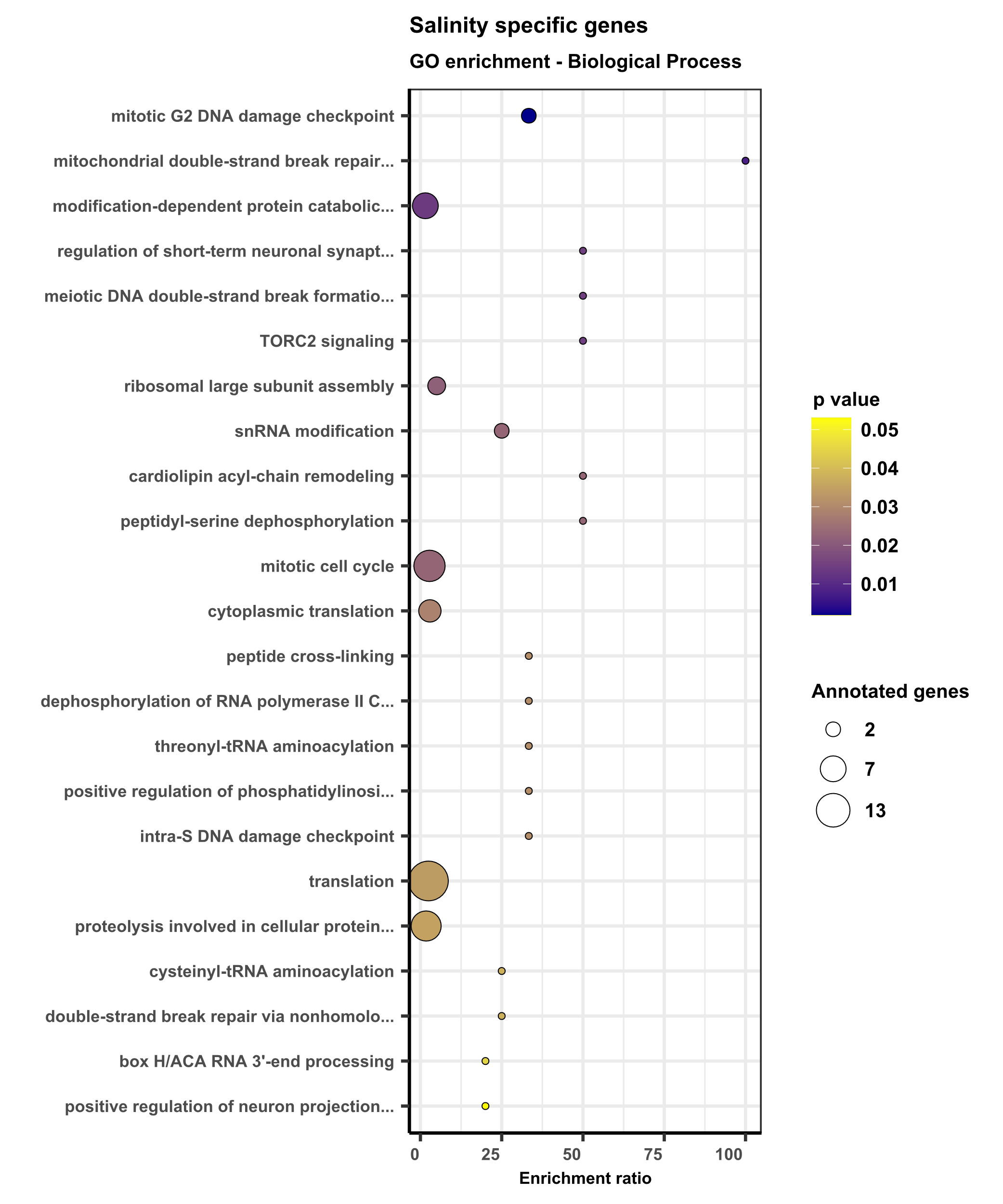


Fig. S1: Biological process term enrichment results for 442 genes exclusively regulated in response to salinity treatments. Enrichment ratio refers to the proportion of observed expressed genes annotated with a GO term, divided by the expected number of genes annotated with the GO term at random. P-values were obtained from Fisher's Exact test and only results for p values < 0.05 are shown.


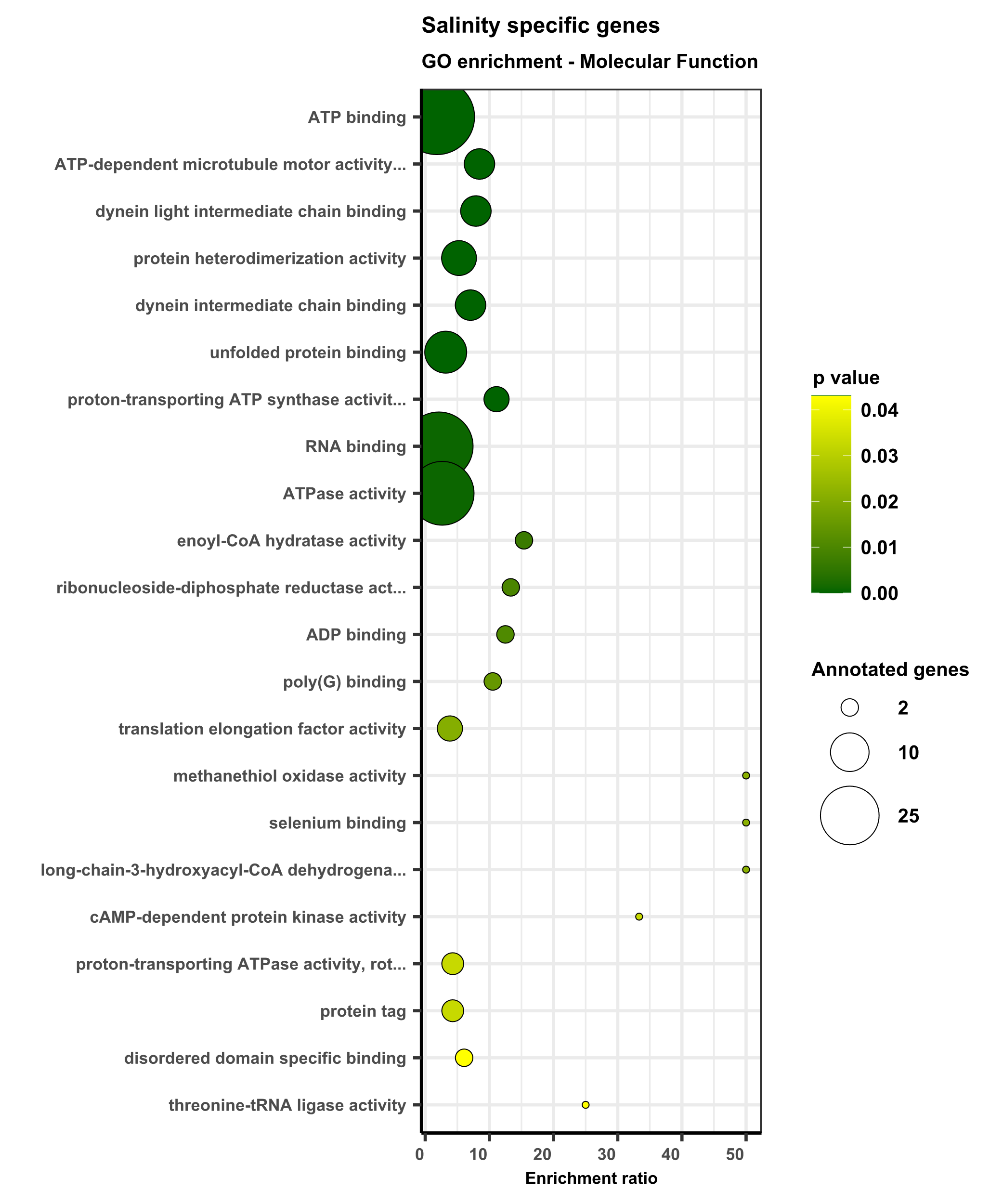


Fig. S2: Molecular function term enrichment results for 442 genes exclusively regulated in response to salinity treatments. Enrichment ratio refers to the proportion of observed expressed genes annotated with a GO term, divided by the expected number of genes annotated with the GO term at random. P-values were obtained from Fisher's Exact test and only results for p values < 0.05 are shown.
